# The Role of RNA Interference (RNAi) in Arbovirus-Vector Interactions

**DOI:** 10.3390/v7020820

**Published:** 2015-02-17

**Authors:** Carol D. Blair, Ken E. Olson

**Affiliations:** Arthropod-borne and Infectious Diseases Laboratory, Department of Microbiology, Immunology & Pathology, Colorado State University, Fort Collins, CO 80523, USA; E-Mail: kenneth.olson@colostate.edu

**Keywords:** RNA interference, exogenous siRNA, piRNA, antiviral defense, innate immunity, transgenic mosquito

## Abstract

RNA interference (RNAi) was shown over 18 years ago to be a mechanism by which arbovirus replication and transmission could be controlled in arthropod vectors. During the intervening period, research on RNAi has defined many of the components and mechanisms of this antiviral pathway in arthropods, yet a number of unexplored questions remain. RNAi refers to RNA-mediated regulation of gene expression. Originally, the term described silencing of endogenous genes by introduction of exogenous double-stranded (ds)RNA with the same sequence as the gene to be silenced. Further research has shown that RNAi comprises three gene regulation pathways that are mediated by small RNAs: the small interfering (si)RNA, micro (mi)RNA, and Piwi-interacting (pi)RNA pathways. The exogenous (exo-)siRNA pathway is now recognized as a major antiviral innate immune response of arthropods. More recent studies suggest that the piRNA and miRNA pathways might also have important roles in arbovirus-vector interactions. This review will focus on current knowledge of the role of the exo-siRNA pathway as an arthropod vector antiviral response and on emerging research into vector piRNA and miRNA pathway modulation of arbovirus-vector interactions. Although it is assumed that arboviruses must evade the vector’s antiviral RNAi response in order to maintain their natural transmission cycles, the strategies by which this is accomplished are not well defined. RNAi is also an important tool for arthropod gene knock-down in functional genomics studies and in development of arbovirus-resistant mosquito populations. Possible arbovirus strategies for evasion of RNAi and applications of RNAi in functional genomics analysis and arbovirus transmission control will also be reviewed.

## 1. Introduction

RNA-mediated gene silencing is an evolutionarily-conserved mechanism in which the presence of intracellular double-stranded (ds)RNA triggers a pathway that leads to the production of small RNAs that mediate regulation of expression (usually inhibition) of genes with cognate sequences to the RNA trigger [1]. RNA interference (RNAi) is a term coined by Fire *et al.* in 1998 [2] when they discovered that injection of dsRNA into the nematode *Caenorhabditis elegans* resulted in potent and specific silencing of expression of the endogenous gene homologous to the RNA. Shortly thereafter, it was demonstrated that dsRNA-mediated gene silencing was also effective in *Drosophila melanogaster* (fruit fly) embryos [3]. In 2000, Baulcombe [4] pointed out that the phenomena known as post-transcriptional gene silencing (PTGS) and virus-induced gene silencing (VIGS), described in plant research from the early 1990s [5,6] are manifestations of a plant anti-viral defense system that uses the dsRNA-triggered RNAi mechanism to inactivate viral RNA. Pioneering plant research also revealed the presence in primed plant cells of ~25 nt antisense RNAs thought to be specificity determinants of PTGS/VIGS/RNAi [7] and the observation that various viruses encode suppressors of gene silencing as counterdefenses to RNAi [8]. RNAi as a defense mechanism against viral or genomic parasites is broadly-distributed in invertebrates and plants; because of the close evolutionary relationship between *Drosophila* and mosquitoes, the fly has become a highly-studied model organism for understanding vector RNAi and its role in arbovirus infections.

The designation RNAi includes at least three pathways that are mediated by small (20–30 nt) RNAs. The pathways are named for the small RNA mediators that are the end-product of each: small interfering (si)RNA, micro (mi)RNA and Piwi-interacting (pi)RNA [9]. The precursor(s) of small RNA in each pathway, the proteins required for small RNA biogenesis and function, and the mechanisms by which each pathway regulates gene expression are varied. The distinct roles of these pathways are defense of the cell and organism against virus infection, regulation of development and gene expression, and defense of the genome against transposon mobilization and expression, respectively, although cross-talk between pathways has been noted in *Drosophila* [9]. The role of the exo-siRNA pathway in arbovirus-vector interactions has been most thoroughly studied; however, recent research has begun to shed light on potential roles of the piRNA and possibly miRNA pathways in vector modulation of arbovirus infections.

## 2. The Exo-siRNA Pathway is an Antiviral Defense Mechanism in Arthropods

Following the discovery of exogenous dsRNA-initiated gene silencing in *Drosophila* [3], extensive research to characterize the components and mechanisms underlying RNAi has been carried out in intact flies, *Drosophila* cell culture lines, and embryo lysates. *Drosophila* is an excellent model organism for this research because of the availability of its complete, assembled, well-annotated genome sequence [10,11] and genetics systems for facile induction and analysis of mutations. For comprehensive reviews of *Drosophila* RNAi see [12,13,14].

The exogenous (exo-)siRNA pathway can be initiated in *Drosophila* not only by injection of dsRNA but also by intracellular generation of foreign dsRNA during virus replication [15]. Endogenous (endo)-siRNAs can arise from dsRNAs created by convergent transcripts or transcription of structured genomic loci [16,17]. Endo-siRNAs are processed by the same pathway components as exo-siRNAs, except that an isoform of Loquacious serves as the dsRNA-binding protein, rather than R2D2 [18]. The role of the exo-siRNA pathway in the *Drosophila* antiviral response has been studied both with insect-only viruses that naturally infect flies and with arboviruses, which can be adapted to infect the fruit fly. Although *Drosophila* can be infected with arboviruses, they are not vectors for arbovirus transmission and only certain aspects of arbovirus-vector interactions can be modeled. Experimental infections in the fly are initiated by intrathoracic injection of high viral doses and acute infections with insect-only viruses result in pathogenesis, particularly if the RNAi response is defective [19]. In contrast, experimental arbovirus infections of mosquitoes can be initiated by the natural route, that is, through an infectious blood meal, and despite the requirement for a high experimental virus dose, both experimental and natural mosquito infections are generally non-pathogenic and persistent.

While it is clear that components of several canonical *Drosophila* innate immune pathways are transcriptionally-induced after virus injection [20,21,22,23], it has been amply demonstrated that the exo-siRNA pathway is the most potent, broadly-reactive antiviral immune response in the fly [19,24,25]. The exo-siRNA pathway is known to be triggered by detection of long dsRNA, which does not normally occur in uninfected insect cell cytoplasm and is thus recognized as a pathogen-associated molecular pattern (PAMP). The initiator dsRNA may be a replicative intermediate formed during replication of a positive-sense RNA viral genome [26,27], as shown by equal proportions of positive- and negative-sense siRNAs derived from the entire length of these viral genomes, or a dsRNA viral genome [24]. Even though little dsRNA is detectable in cells infected with negative-strand RNA viruses [28,29], the findings in both fruit fly and mosquito cells that siRNAs derived from the genomes of negative-sense RNA viruses such as vesicular stomatitis virus (VSV, *Vesiculovirus*), La Crosse virus (LACV, *Orthobunyavirus*), and Rift Valley fever virus (RVFV, *Phlebovirus*) map to the entire genome in both positive- and negative-sense polarity, suggest that they are derived from duplexes formed during replication or transcription of the virus genome [29,30,31]. Recent characterization of precursors of virus-specific siRNAs in *Drosophila* cells demonstrated that exo-siRNAs, like endo-siRNAs, can also be derived from intramolecular secondary structures such as stem-loops in viral RNA, self-complementary fold-back regions of defective-interfering RNA, transcripts from tandem-repeat non-coding sequences in a mammalian DNA virus genome and overlap regions of convergent transcripts from insect DNA virus genomes [25,32,33].

The RNase III family nuclease Dicer 2 (Dcr2) acts as the pattern recognition receptor (PRR) for the exo-siRNA pathway, detecting long dsRNA and cleaving it to siRNAs consisting of two ~21–22 nt base-paired strands with 5'-phosphorylated ends and 2-nt single-strand overhangs on the 3'-hydroxyl ends [19,34]. These remain complexed with Dcr2, which interacts with the dsRNA-binding protein R2D2 to load the siRNA into the RNase H family endonuclease Argonaute 2 (Ago2) [35,36]. Ago2 is the active component of a multi-protein RNA-induced silencing complex (RISC) and it first cleaves the siRNA “passenger” strand, retaining the “guide” strand for identifying and hybridizing to cytoplasmic ssRNA such as viral mRNA with the precise complementary sequence. Ago2 catalyzes cleavage, or “slicing”, of the phosphodiester bond of the RNA target between nts 10 and 11 of the hybridized siRNA [36,37,38].

Events or functions downstream of vsiRNA production by Dcr2 and Ago2 cleavage of arboviral RNA that might be required for or enhance effective control of virus replication are not well-characterized; however, several observations in *Drosophila* suggest that further activities might be significant. Virus genome cleavage generates two RNA fragments; the 5'-end fragments are degraded from their 3'-ends by the exosome and the 3′-end fragments are degraded from their 5′-ends by the exonuclease XRN1 [39]. XRN1 is required for degradation of mRNAs targeted by RNAi in *Drosophila* [40].

A *Drosophila* gene termed *vago* encodes a small secreted protein that was shown to control replication of drosophila C virus in the fly’s fat body. Induction of Vago expression was shown to be dependent on the *N*-terminal DExD/H-box helicase domain of Dcr2 and independent of the *C*-terminal RNase domains [41]. The authors of this study suggested that, like the phylogenetically related RIG-I-like receptors that trigger type I interferon induction in mammals, Dcr2 of insects represents an evolutionarily conserved set of PRRs that induce a coordinated antiviral response [41]. The study did not determine the signaling pathway for Vago induction or identify a role for Dcr2 in induction of other possible antiviral functions. Paradkar *et al.* [42,43] have described a *Culex quinquefasciatus* ortholog of *vago* that also depends on Dcr2 induction and restricts flavivirus replication in mosquitoes.

RNAi in *C. elegans* and plants is a systemic antiviral response that protects both local and distal tissues when long dsRNAs or vsiRNAs that are generated in infected cells are amplified by cellular RNA-dependent RNA polymerase (RdRP) and released to spread to and be taken up by uninfected cells [44]. RNAi in insects was assumed to be a cell-autonomous response [45]. However, Saleh and colleagues [46] identified a pathway for uptake of long dsRNA in *Drosophila* cells that involved recognition of dsRNA by scavenger receptors [47], followed by endocytosis, which was essential for effective antiviral RNAi mediated by the exo-siRNA pathway [48].

Although experimental acute infections of *Drosophila* are initiated by intrathoracic injection of a high virus dose and frequently result in host mortality, most natural infections are persistent and exact no fitness cost to the fly. Using an experimental Flock House virus-Drosophila model, Saleh and colleagues [49] showed that early in infection, fragments of the RNA virus genome were reverse-transcribed by retrotransposons and the resulting cDNAs were integrated into the *Drosophila* genome embedded in reverse transcripts of the retrotransposon. RNAs transcribed from these nuclear DNA chimeras were processed in the cytoplasm by the RNAi machinery to produce vsiRNAs that inhibited viral replication. This balance between virus replication and vsiRNA production provided a mechanism for natural viral persistence. Importantly, in the absence of a *Drosophila*-encoded RdRP, the structured transcripts from virus genome-derived sequences in *Drosophila* DNA also provided a potential source of long dsRNA that could be released to mediate a systemic RNAi response [49].

Although identification of long dsRNA as the trigger for RNAi was first accomplished in worms and flies in 1998, emulation of the earlier studies of plant virologists led us to the discovery in 1996 that RNA-mediated gene silencing could be engineered in mosquitoes to interfere with arbovirus replication. We demonstrated that expression from the recombinant RNA genome of a Sindbis virus (SINV, *Alphavirus*) transducing vector of a fragment of the heterologous viral genome or anti-genome RNA could trigger virus-induced gene silencing (VIGS) to inhibit LACV or dengue virus (DENV, *Flavivirus*) replication in mosquito cell cultures and *Aedes aegypti* [50,51,52]. We later showed that production in mosquito cells of dsRNA of 150 bp or longer derived from DENV genome sequence was the trigger for mosquito cell RNAi [53,54,55].

Publication of the complete genome sequences of the mosquitoes *Anopheles gambiae* in 2002 [56], *Aedes aegypti* in 2007 [57], and *Culex quinquefasciatus* in 2010 [58], enabled identification of orthologs of *Drosophila* RNAi pathway genes in each of these vector species [34] and characterization of essential components of the antiviral response. We identified and knocked down expression of *dicer* and *argonaute* genes in *An. gambiae* by RNA-mediated gene silencing (intrathoracically injecting dsRNA derived from each gene) during infection with o’nyong nyong virus (ONNV, *Alphavirus*), the only arbovirus known to be transmitted by *An. gambiae* [59]. Knocking down expression of Ago2 resulted in production of 16-fold higher infectious ONNV titers than in mosquitoes injected with an irrelevant dsRNA, demonstrating that RNAi acts as a potent mosquito anti-viral defense and that components of the exo-siRNA pathway are essential [59]. Silencing Dcr2 and Ago3 expression also resulted in significantly higher ONNV titers (see Section 3). Although research on RNAi in *Anopheles* spp. has not been extensively pursued, a recent study in which *An. gambiae* were infected by imbibing an ONNV-containing blood meal suggested that exo-siRNA suppression of virus replication was more important after dissemination to other tissues of the mosquito than in the midgut [60].

Publication of the *Ae. aegypti* genome sequence similarly allowed us to show that Dcr2, Ago2 and R2D2, key components of the exo-siRNA pathway, are required for robust antiviral defense against dengue virus type 2 (DENV2) in this important vector. In particular, silencing of Dcr2 resulted in a 10-fold increase in DENV2 titer and a reduction in the extrinsic incubation period from 10 days to 7 days [61]. We also demonstrated the appearance after DENV infection of small RNAs derived from both the genome-sense and anti-sense viral RNA with a size consistent with siRNAs. We pursued characterization of DENV-specific siRNAs (vsiRNAs) by next generation (NexGen) sequencing and determined that DENV2-infected *Ae. aegypti* cultured cells (Aag2) and *Ae. aegypti* mosquitoes produced a population of vsiRNAs that mapped to the entire viral genome, contained approximately equal proportions of sense (genome)-strand and anti-sense reads and were predominantly 21 nt in length [62], properties suggesting that they were the products of Dcr2 cleavage of replicating DENV RNA. Importantly, we showed that C6/36 *Ae. albopictus* cells exhibit defective Dcr2 cleavage of DENV RNA because of a single nucleotide deletion in their *dicer2* gene, and DENV RNA-derived small RNAs in these cells have properties suggestive of piRNAs (see Section 3). The defective Dcr2 activity is associated with DENV2 growth to 10- to 100-fold higher titers in C6/36 cells than in RNAi-competent Aag2 cells, emphasizing the importance of the siRNA pathway in mosquito antiviral defense [62]. We also found that piRNA-like virus genome-derived small RNAs, rather than siRNA-like small RNAs, were produced in C6/36 cells infected with the mosquito-only cell-fusing agent virus (CFAV, *Flavivirus*) [62], SINV and LACV [30].

A number of other studies have demonstrated and characterized the antiviral exo-siRNA response to infection by arboviruses from diverse genera (*Flavivirus*, *Alphavirus*, *Orthobunyavirus*, *Phlebovirus*, *Vesiculovirus*) in cultured cells from *D. melanogaster*, *Ae. albopictus*, and *Ae. aegypti* [29,31,63] and in adult *D. melanogaster* and *Ae.* *aegypti*, *Ae. albopictus* and *Cx. quinquefasciatus* mosquitoes [19,64,65,66,67,68]. In addition, other arthropod vectors have been found to exhibit an RNAi response to arbovirus infection. Schnettler *et al.* [69] showed that cultured cells derived from *Culicoides sonorensis* midges, after infection with either bluetongue virus (*Orbivirus*) or Schmallenberg virus (*Orthobunyavirus*), produced 21 nt vsiRNAs with profiles suggesting production by a Dicer-like nuclease. Availability of the genome sequence of the tick *Ixodes scapularis* in VectorBase [70,71] has allowed identification of *dicer* and *argonaute* genes related to those of RNAi pathway genes of insects [72] and gene silencing by dsRNA has been demonstrated in tick cell lines [73]. A Langat virus (*Flavivirus*)-infected *I. scapularis* cell line produced vsiRNAs with an average 22 nt length [74].

The exo-siRNA pathway has been shown to be an important and diverse antiviral response of several arthropod vectors to infection by arboviruses from a number of families. Arboviruses from different families, thus with different genome structures, elicit exo-siRNA responses with differences in magnitude, time of appearance, and size distribution and polarity of small RNAs, even in the same vector (e.g., *Ae. aegypti* infected with flaviviruses *vs.* alphaviruses).

## 3. The piRNA Pathway and Arbovirus-Vector Interactions

The Piwi-interacting (pi)RNA pathway is more recently discovered in arthropods and not as well characterized as the siRNA and miRNA pathways. In *Drosophila*, piRNAs are 24–30 nt long and regulate transposable element transcription and transposition [75,76]. The pathway components were thought to be expressed exclusively in germ-line cells for protection of the genome, but are now known also to be functional in at least some somatic cells of both *Drosophila* and mosquitoes [68,77,78]. Three proteins have been uniquely implicated in piRNA biogenesis in *Drosophila*, Piwi, Aubergine (Aub) and Ago3; all are members of the Piwi subfamily of the Argonaute protein family. No Dicer-family genes are involved in piRNA biogenesis. Primary piRNA precursors are transcribed by RNA polymerase II from discrete genomic loci that are mostly derived from defective transposon sequences [75,76]. Piwi and Aub are usually bound to piRNAs that are antisense to retrotransposon transcripts and tend to have a 5'-uridylic acid (U_1_) residue. Ago3 binds to complementary positive-sense piRNAs that usually have an A at position 10 (A_10_). These complementary relationships have suggested a “ping-pong” generation and amplification loop in which each piRNA-directed cleavage event by one of the Piwi proteins generates the 5'-end of a new piRNA.

*An. gambiae* have *ago3* genes that are orthologous to *Drosophila ago3* and two additional *piwi* subfamily genes, *ago 4* and *ago 5* [34]. By comparison to *Drosophila*, the *piwi* subfamily genes of Aedine mosquitoes have undergone expansion during evolution. *Ae. aegypti* have an *ago3* and *piwi1-7* genes and *Cx. quinquefasciatus* have an *ago3* and *piwi1-6* genes. None of the mosquito *ago* or *piwi* genes is orthologous to either *Drosophila* *aub* or *piwi* genes [34].

Wu *et al.* [77] first described virus genome-derived piRNAs in *Drosophila* ovary somatic sheet cell cultures. Several recent studies have revealed the presence of virus genome-specific piRNAs in infected mosquito cells [30,31,62,68,79,80]. In DENV2-infected, Dcr2-defective C6/36 cells, the predominant (96%) virus RNA-derived small RNAs were 27 nt long, had genome (positive)-sense polarity, and a bias for A_10_, in contrast to the predominant vsiRNAs that were 21 nt long, had equal positive-negative polarity, and no nucleotide bias at any position in Dcr2-competent Aag2 cells and *Ae. aegypti* [62]. Thus, the virus-derived small RNAs in flavivirus-infected C6/36 cells had piRNA-like properties, but their mechanism of generation was not determined. However, C6/36 cells infected with either the alphavirus SINV or the bunyavirus LACV generated piRNA-like virus-specific small RNAs with a positive strand bias that had the signature of ping-pong amplification [30,80]. In addition, *Ae. albopictus* mosquitoes infected with chikungunya virus (CHIKV, *Alphavirus*), SINV-infected U4.4 and Aag2 cell cultures, and RVFV-infected Aag2 cell cultures all produced both virus RNA-derived siRNAs and piRNAs with ping-pong cycle signatures [31,68,80]. Interestingly, in RVFV-infected Aag2 cells, 21 nt vsiRNAs predominated during early (acute) infection whereas 24–27 nt vpiRNAs were predominant by 72 h when viral persistence was established [31].

The mechanism by which vpiRNAs are generated and which of the proteins in the Piwi subfamily are involved in their production is not clear, although Ago3 was implicated in *An. gambiae* defense against an ONNV infection [59] and Piwi4 was suggested to play a part in inhibiting Semliki Forest virus (SFV, *Alphavirus*) replication in *Ae. aegypti* (Aag2) cell cultures [81]. In an interesting comparison of piRNAs in uninfected *Drosophila* and *Ae. aegypti*, Arensburger *et al.* [82] found that, while 15.8% of the 144 Mb sequenced genome of *D. melanogaster* was composed of transposons, 51% of piRNA sequences were transposon-derived. In contrast, 47% of the 1.38 Gb genome of *Ae. aegypti* is composed of transposons, but only 19% of its piRNAs mapped to transposons. Many *Ae. aegypti* piRNAs mapped to coding sequences, including a number of endogenous virus genome-related sequences. Arensburger *et al.* suggested that the functions of the piRNA pathway might differ between *Ae. aegypti* and *D. melanogaster*, with defense of the genome against virus as well as transposon invasion having greater importance in the vector mosquito.

Whether or not virus genome-derived piRNAs play a role in vector antiviral defense requires further investigation. The piRNA pathway might complement the exo-siRNA pathway, compensate for a defective siRNA pathway, or have major responsibility for control of persistent virus infection, depending upon the virus-vector combination and functionality of RNAi pathway genes. These and other questions remain open.

## 4. Does the miRNA Pathway Have a Role in Arbovirus-Vector Interactions?

The major role of the miRNA pathway in all metazoans and plants is regulation of development and gene expression; whether or not miRNAs or the miRNA pathway play specific roles in arbovirus-vector interactions has not been extensively explored. Canonical miRNA biogenesis is triggered by transcription of primary miRNAs (pri-miRNA) from miRNA clusters in the *Drosophila* genome by RNA polymerase II [83]. Pri-miRNAs are cleaved by a nuclear RNase III nuclease called Drosha to pre-miRNAs consisting of imperfectly base-paired stem-loops of 60–70 nt. After Exportin 5-dependent transport of pre-miRNAs to the cytoplasm, Dicer 1 (Dcr1) and its associated dsRNA-binding protein Loquacious (Loq) recognize the 2-nt 3'-end overhangs on pre-miRNAs and cleave them to produce imperfectly base-paired miRNA duplexes, each consisting of a ~22–23 nt miRNA guide strand and miRNA* passenger strand [84]. The guide strand is loaded into Argonaute 1 (Ago1), which is a component of a RISC that directs limited base-pairing, usually to the 3'-untranslated region (UTR) of a mRNA, through a “seed region” of ~8 nt at the 5'-end of the miRNA [85]. Binding of the miRNA-containing RISC suppresses translation or leads to degradation of the mRNA. miRNAs can also be generated by non-canonical mechanisms from short hairpin introns, using the spliceosome rather than Drosha to cleave their precursor ends [86,87], and from RNA polymerase III transcripts of retroviral DNA proviruses [88]. At least 466 distinct *D. melanogaster* miRNAs are available in miRBase [89] and mechanisms for their biogenesis, editing and evolution have been described [90,91,92]. Cloning and deep sequencing have identified conserved miRNAs in mosquito vectors as well as a number that are found only in mosquitoes [93,94,95,96].

An in-depth review of the role of miRNAs in arbovirus-vector interactions has been published recently [97], so we will touch on the subject here only as it relates overall to vector RNAi. Although some mammalian viruses are known to activate or repress expression of cellular miRNAs to facilitate replication [98,99], only a few publications have suggested that arboviruses have exploited this mechanism. Several studies have shown that levels of expression of mosquito miRNAs are modulated after blood-feeding and/or arbovirus infection, and in some cases, particular miRNAs have been identified that appear to enhance [100] or restrict [101] flavivirus replication in *Aedes* spp. mosquitoes or cultured cells by altering canonical immune mechanisms. Campbell *et al.* [102] conducted a thorough analysis of small RNA library sequence data from DENV2-exposed *Ae. aegypti* to examine changes in miRNA expression levels after infection. Although only 50% of the infectious blood-meal-fed mosquitoes acquired a DENV infection, by nine days post exposure they exhibited significant changes in levels of expression of 23 unique miRNAs as compared to mosquitoes that had imbibed a non-infectious blood-meal, and only three of these miRNAs showed enhanced expression levels. Putative 3'-UTR binding sites for these miRNAs were predicted in mRNAs of 464 genes encoding proteins involved in transport, transcriptional regulation, mitochondrial function, chromatin modification and signal transduction processes. These were linked to previous studies defining host factors involved in DENV replication, but no speculations regarding specific functions were made.

In an interspecific study of *Drosophila*, genes encoding components of the exo-siRNA pathway were found to be among the fastest evolving 3% of all genes. In contrast, paralogous miRNA pathway genes did not evolve more rapidly than the rest of the genome [103]. *Drosophila*-pathogenic viruses bear genes encoding suppressors of antiviral exo-siRNA (see Section 5), which also evolve rapidly, suggesting that infection with pathogenic RNA viruses may drive an evolutionary arms race between virus and host [104]. We examined the comparative patterns of molecular evolution between the exo-siRNA and miRNA pathway genes in six geographically-distinct populations of *Ae. aegypti*. We analyzed the genetic diversity of *dicer1*, *argonaute1* and *r3d1 (loqs)* from the miRNA pathway and *dicer2*, *argonaute2* and *r2d2* from the exo-siRNA pathway of these mosquitoes and found, surprisingly, that both exo-siRNA and miRNA pathway genes appear to be undergoing rapid, positive, diversifying selection [105]. Wild *Ae. aegypti* populations have a low DENV infection prevalence (Yoon 2012 [106]), DENV infection has little fitness cost for *Ae. aegypti* [107], and DENVs are not known to encode potent protein suppressors of exo-RNAi (see Section 6); thus we would not expect a similar DENV-driven pattern of evolution in the *Ae. aegypti* exo-siRNA pathway genes. Our results could be interpreted to suggest a role for both the miRNA and siRNA pathways in antiviral defense; however, they might also signify interactions between components of the RNA silencing pathways for other reasons, as was shown, for example, by formation of Dcr2-Loqs heterodimers for loading endo-siRNAs into Ago2-RISC for suppression of transposon activity in *Drosophila* [16].

A number of vertebrate DNA viruses and retroviruses are known to encode in their genomes unique miRNAs that regulate viral gene expression or have roles in evasion of mammalian innate immune responses [108]. Precursors of many of these viral-encoded miRNAs are transcribed in the nucleus by host cell RNA polymerases II or III and exported to the cytoplasm by mechanisms that do not involve Drosha excision of the pre-miRNA from the viral mRNA or genome [88]. There have been two reports of miRNAs encoded in the 3'-UTRs of flavivirus genomes [109,110]; however, comments from other investigators [111,112] have raised questions about the general applicability of these findings.

Clearly, further research is needed to define and characterize the roles of the miRNA pathway and miRNAs in arbovirus-vector interactions.

## 5. Arboviral Evasion of RNAi

All plant viruses and insect-pathogenic viruses that have been examined encode protein suppressors of RNAi (called viral suppressors of RNAi, VSRs) that act by diverse mechanisms to directly block RNAi-based restrictions on viral replication [113,114]. Unrestrained replication by these viruses usually leads to pathogenesis and VSRs are thus considered virulence factors [115]. Similar arbovirus-encoded protein VSRs have not been identified. Arbovirus infections of mosquito cells are generally nonpathogenic, and robust suppression of RNAi resulting in pathogenesis and death of the vector would be detrimental to maintenance of the natural virus transmission cycle [65,66]. Nevertheless, it is probable that arboviruses have evolved more subtle mechanisms for evasion of mosquito RNAi in order to establish a balance between killing the host and being completely cleared by its potent antiviral defense. Various mechanisms have been proposed for arboviral evasion of RNAi, but evidence for any avoidance strategy is incomplete.

One potential mechanism of RNAi evasion is sequestration within membrane vesicles of the dsRNA PAMP that triggers exo-siRNA. For example, electron tomographic reconstruction to reveal three-dimensional rearrangement of intracytoplasmic membrane structures in mosquito cells after DENV infection showed that dsRNA-containing replication complexes were enclosed in double-membrane vesicles [116], as had been shown previously for mammalian cells [117].

Because viral RNA replication results in accumulation of high virus-specific RNA concentrations in infected cells, it has been proposed that abundant viral RNAs may serve as decoys or “sponges” to sequester cellular RNA-binding proteins and thus reduce their ability to carry out normal, possibly anti-viral, functions [118]. A potential RNAi decoy or sequestration mechanism has been proposed for the subgenomic flavivirus RNA (sfRNA), a highly-structured, ~300–500 nt RNA derived from the genome 3'-UTR and produced in all cells infected with mosquito- and tick-borne flaviviruses [119]. The sfRNA is produced when the cellular 5'-to-3' exoribonuclease XRN1 stalls at the conserved 5'-end of the highly-structured flavivirus 3'-UTR. It has been shown that XRN1 stalling on sfRNA inhibits and inactivates this enzyme in both mammalian and mosquito cells, an activity that may be required to complete viral genome degradation following Ago2 cleavage in arthropod cells [120]. XRN1 sequestration may thus stall the RNAi process. It has also been proposed that WNV sfRNA acts as a competing substrate for Dicer in insect cells on the basis of its inhibition of recombinant human Dicer cleavage of long dsRNA in an *in vitro* assay [121].

The possibility that the arbovirus RNA genome undergoes mutations to prevent complementary base-pairing to the cleavage target by Ago2-associated vsiRNAs is suggested by other studies. In most reports in which deep-sequencing was used to characterize arbovirus genome-derived siRNAs, it has been noted that vsiRNAs are asymmetrically distributed along the length of the virus genome, with more intensely targeted regions (hot-spots) and other areas with no or low frequency generation of vsiRNAs (cold-spots). Brackney *et al.* [67] found in West Nile virus (WNV; *Flavivirus*)-infected *Cx. quinquefasciatus* that WNV RNA hot-spots are more likely to contain point mutations compared to cold-spots. Siu *et al.* [63], in a study of SFV-infected U4.4 (*Ae. albopictus*) cells, prepared synthetic vsiRNAs and found that hot-spot vsiRNAs were significantly less efficient at mediating antiviral RNAi than cold-spot viRNAs. They suggested that this finding pointed toward a viral genome-based decoy mechanism to evade the RNAi response.

Intriguing observations have been made of possible altered functions of the components of the exo-siRNA pathway in *Drosophila* cells during persistent viral infection. The insect-only nodavirus Flock House virus (FHV) has been extensively used in model studies of *Drosophila* antiviral RNAi. Acute FHV infection in *Drosophila* S2 cells is highly cytopathic and is lethal in adult flies [122]. During acute infection of S2 cells, typical 21-nt vsiRNAs are produced, but they are bound by the virally-encoded B2 protein, a VSR, and thus are ineffective in controlling infection [26]. Acute infections by FHV with a deleted B2 gene (FHVΔB2) can be terminated by *Drosophila* RNAi, requiring the activities of Dcr2, R2D2 and Ago2 [122]. Surprisingly, it was found that the S2 cell lines maintained in several laboratories were persistently-infected with FHV that retained full infectivity for uninfected cells and had a functional B2 protein. Furthermore, Dcr2 and Ago2 were shown to be active and abundant and typical 21-nt vsiRNAs were generated in the persistently infected cells [27]. However, only a minor fraction of vsiRNAs were associated with Ago2-RISC, suggesting that assembly of functional silencing complexes was impeded and that Dicer cleavage of viral replication intermediates had an important role in controlling virus replication to maintain persistence [27]. Whether alteration of expression or function of mosquito exo-siRNA pathway components occurs with establishment of persistent arbovirus infection has not been examined; however, interestingly, Léger *et al.* [31] observed that RVFV genome-derived small RNAs were predominantly 21-nt Dcr2 siRNAs during early infection of both Aag2 and U4.4 mosquito cells and 24- to 27-nt piRNAs became predominant as the infection became persistent, suggesting a possible role for vpiRNAs in control of infection as the exo-siRNA response wanes.

## 6. RNAi as a Tool in Functional Genomics of Vectors

Since the discovery that the presence of exogenous intracellular dsRNA could trigger silencing of expression of homologous genes [2,3], RNAi has been used as a tool to examine the effects of transient knock-down of expression of specific vector genes on replication of arboviruses. An efficient method for delivery of intracellular dsRNA to either cultured cells or adult mosquitoes has been by use of double subgenomic alphavirus transducing vectors, such as SINV-TE3'2J, in which a 100–500 nt RNA fragment from the gene of interest can be inserted into the genome of a recombinant alphavirus under the control of a duplicated subgenomic promoter [51,123,124]. The virus readily infects most mosquito cells and expresses the inserted RNA fragment as part of its dsRNA replicative intermediate. The dsRNA may also be expressed as a transcript from an inverted repeat (IR) sequence cloned into a transforming plasmid or transposable element [55,125]. The dsRNA also is frequently prepared by *in vitro* transcription of both strands of a cDNA clone prepared from the mRNA or virus genome of interest, followed by either intrathoracic inoculation into mosquitoes [59] or soaking or use of a transfection reagent for introduction into the cytoplasm of cultured cells [62,126].

Transient knock-down of expression of genes in the various RNAi pathways has been an extremely important tool in defining essential components of antiviral defense in mosquitoes [22,59,61,81]. The tool has also been used more widely to define specific host factors with potential roles in virus replication [127] or in genome-wide screens to broadly identify host factors that modulate arbovirus infections in cultured *Drosophila* cells [128,129].

## 7. Harnessing Mosquito RNAi to Reduce Arbovirus Transmission

The antiviral exo-siRNA response in mosquitoes suggests novel ways of interfering with arbovirus replication in the vector. A strategy was described some years ago, first in plants and then *Drosophila* for expressing inverted repeat (IR) RNA sequences in sense and antisense orientations. The transcript of the IR sequence forms intracellular dsRNA to silence specific host genes [130,131]. Using a similar strategy to target DENV2 instead of a host gene, we generated *mariner* (*Mos1*)-transformed *Ae. aegypti* lines to express DENV2-specific dsRNA in the mosquito midgut [125,132]. In the transgenic mosquitoes, expression of the dsRNA was under control of the *Ae. aegypti* *carboxypeptidase A* promoter and occurred in midgut epithelial cells within hours of the mosquito acquiring a blood-meal. We screened a number of generation 3 (G_3_) transgenic lines for their ability to silence DENV2 replication. One of these lines, called Carb109M, was highly efficient at silencing DENV2 replication in the midgut, making the mosquito line refractory to infection [132]. Genetic analyses and physical chromosome mapping identified two closely linked transgene integration sites in Carb109M near the gene encoding a polyadenylate binding protein, mapping terminally at 70 centimorgans (cM) in chromosome 3. Northern blot analysis detected abundant, transient expression of the IR-RNA 24 h after a blood-meal. NexGen sequencing of midgut small RNAs from blood-fed, but uninfected, Carb109M revealed that the IR-RNA was rapidly processed into 21-nt small RNAs with sequences corresponding to the IR-RNA target region of the DENV2 RNA genome (prM/M coding region). Expression of a DENV2 sequence-derived IR-RNA in the mosquito midgut initiated the antiviral intracellular RNAi response early in the initial site of infection, efficiently blocking DENV2 infection and profoundly impairing vector competence for DENV2. Carb109M mosquitoes were refractory to infection with various DENV2 genotypes, but not to other DENV serotypes due to the sequence specificity of the exo-siRNA pathway. The two transgene integration sites were stable after multiple generations (G_33_) and following introgression into a second *Ae. aegypti* population from Mexico (GDLS strain). Introgression of the transgene into GDLS having a different genetic background from Carb109M changed the GDLS population from a highly DENV2-permissive phenotype to a DENV2-refractory phenotype. Significantly, the DENV2-refractory homozygous line, Carb109M/GDLS.BC5.HZ, exhibited (relative to GDLS) minimal fitness loss associated with the transgene [132].

Transgenic *Ae. aegypti* also have been developed that express resistance to DENV2 in another tissue relevant to transmission. An effective salivary gland-specific, female-specific promoter was used for expression of the DENV2-derived IR-RNA in transformed *Ae. aegypti*. The promoter was originally shown to drive expression of the anti-platelet protein (*AAPP*) gene, a member of the *30K* gene family of *Anopheles stephensi*, in the distal-lateral lobes of female salivary glands [133]. In *Ae. aegypti*, the *30K* promoter homolog controls bi-directional expression of 30 Ka and 30 Kb proteins. The DENV2-specific IR-RNA, driven by the 30 Kb promoter, expressed the anti-DENV2 effector sequence and reduced significantly the prevalence and mean intensities of viral infection in mosquito salivary glands and saliva (Mathur 2010 [134]). This was a demonstration that RNAi-based resistance to arbovirus replication is effective in at least two different mosquito tissues that are relevant to virus transmission by the vector.

A number of challenges remain to be solved before using exo-siRNA-based genetically modified mosquitoes as an effective strategy for significantly reducing DENV and other arbovirus transmission and impacting disease. First, IR transgenes must be developed that target and destroy RNA genomes of all four DENV serotypes and/or multiple other arbovirus types. We are currently designing a tetravalent IR effector gene targeting the conserved NS5 (RdRP) coding region of DENV serotypes 1–4. In transgenic plants, several examples have been described in which RNAi-based approaches have been used to successfully target multiple, genetically distinct Tospoviruses [135,136]. Second, in principle the anti-pathogen gene conferring a DENV-refractory phenotype would require introgression into existing DENV susceptible mosquito populations to replace DENV competent *Ae. aegypti* populations with refractory populations. To accomplish this, the anti-DENV IR effector gene may require linkage with an *Ae. aegypti*-specific selfish genetic-element or gene drive system to enable fixation of the transgene in the target vector population [137]. Killer-rescue based gene drive systems such as *Medea* are currently under development in *Ae. aegypti* [138]; however, gene drive approaches are not likely to be implemented as large-scale public health measures in the near future. Recently, Okamoto *et al.* [139] used a stochastic, spatially explicit model of *Ae. aegypti* populations from Iquitos, Peru, to evaluate whether population replacement is feasible absent gene-drive. The modeling indicated that releasing mosquitoes carrying only an anti-pathogen construct could negatively impact vector competence of a natural population at ratios well below those considered necessary for current transgenic vector technologies designed to reduce vector populations [139,140,141]. Moreover, Okamoto and colleagues found that mosquitoes carrying only an anti-pathogen gene are considerably more robust for immigration into wild-type mosquito populations than other strategies modeled. A third challenge is to determine whether arbovirus quasispecies populations selected by exposure to a population of transgenic mosquitoes expressing RNAi-based immunity will remain susceptible to the heritable RNAi-based strategy. A final challenge is that any genetically modified vector approach will need extensive field testing, encounter regulatory hurdles, and require local and regional consent prior to release of any genetically modified mosquito [142,143].

## 8. Concluding Remarks

The research reviewed here has given us a great deal of basic knowledge of the roles of RNAi in interactions between arboviruses and vectors, but also reveals a number of gaps that need to be filled for a fuller understanding. Among the questions to be addressed are the following:

- What are the molecular/structural requirements for recognition of substrates by Dcr2 to trigger the exo-siRNA pathway?

Deddouche *et al.* [41] showed that *Drosophila* and *Aedes* Dcr2 are DExD/H-box helicases structurally related to RIG-I and MDA-5 of mammals, which are PRRs that recognize cytoplasmic viral RNAs and initiate type I interferon induction, a key antiviral innate immune response. Extensive research has determined the structural features of viral RNA PAMPs that are recognized by RIG-I and MDA-5 (e.g., [144,145,146,147]). Similar research approaches could be used to determine the virus-specific RNA structures recognized by mosquito Dcr to elucidate mechanisms of viral evasion of RNAi and to facilitate development of transgenic virus-resistance strategies.

- What are the molecular or other requirements for vsiRNAs to serve as effective guide strands for Ago2? Are all vsiRNAs loaded into Ago2 with equal efficiency? Do the vsiRNAs in Ago2-RISCs turn over during the course of an arbovirus infection?

Flynt *et al.* [27] showed that *Drosophila* cells persistently infected with Flock House virus contained functional Dcr2 and Ago2; however, only a minor fraction of the abundant vsiRNAs generated in these cells were associated with Ago2-RISC. Mosquito cells persistently infected with an arbovirus should be examined to determine if RISC-loading of vsiRNAs stalls in a similar fashion so that vsiRNAs generated late in infection, when newly synthesized viral genomes have accumulated multiple mutations [148,149,150], may be excluded from RISC, thus precluding cleavage of genetically diverse genomes. This would involve examination of the following question: How much viral genome mutation (quasispecies diversity) can be tolerated without loss of RNAi effectiveness?

- What downstream events are required for most effective antiviral activity of the exo-siRNA response? Do mosquito vectors have the capability for systemic RNAi?

The many studies in which intrathoracic inoculation of long dsRNA was shown to mediate effective RNAi in various mosquito tissues suggest an active mechanism for dsRNA uptake in mosquito cells, but mechanisms have not been demonstrated. Attarzadeh-Yazdi *et al.* [151] demonstrated propagation of antiviral RNAi by cell-to-cell spread of dsRNA in mosquito cell cultures, but systemic RNAi has not been thoroughly explored in intact vectors.

- What strategies do arboviruses employ to evade RNAi?

Arboviruses have not been shown to encode protein suppressors of RNAi. Several studies have suggested that unique viral RNA structures might act as molecular sponges to sequester or inactivate host cell proteins required for antiviral activities in mammalian cells [118] and such activities should be examined in mosquito cells.

What role does diversity of genes encoding components of the RNAi pathways play in the genetics of vector competence for arbovirus transmission? Black and colleagues have mapped several genetic quantitative trait loci (QTL) that control *Ae. aegypti* susceptibility to midgut infection and dissemination of DENV by conducting crosses of susceptible and resistant mosquito lines and challenging with a standard DENV strain [152,153]. Research by Lambrechts and colleagues indicated that vector competence of *Ae. aegypti* for DENV depends to a large extent on specific vector genotype-by-virus genotype (G × G) interactions [154], and challenged three isofemale families derived from field-caught *Ae. aegypti* with three contemporaneous low-passage isolates of DENV. They mapped both QTL dependent on mosquito genotype effect, regardless of virus isolate, and QTL dependent on effects of both mosquito and virus genotypes (G × G) [155]. Although fine mapping of genetic loci within QTL were not conducted in these QTL mapping studies, Lambrechts and colleagues earlier demonstrated in a G × G study that natural polymorphisms in the *Ae. aegypti* *dicer2* gene are associated with DENV isolate-specific resistance [156].

- What is the mechanism of production of virus genome-derived piRNAs in mosquitoes and do vpiRNAs have a role in antiviral defense?
- Does modulation of vector miRNA expression play a role in vector-virus interactions and by what mechanisms?
